# Treatment and related morbidity of nasal cavity and paranasal sinus cancers

**DOI:** 10.3389/fonc.2024.1422892

**Published:** 2024-09-26

**Authors:** Nils Smaadahl, Sara-Lynn Hool, Philipp Reinhardt, Lucas Mose, Ralph Hohenberger, Roland Giger, Daniel Hendrik Schanne, Lukas Anschuetz

**Affiliations:** ^1^ Department of Otorhinolaryngology, Head and Neck Surgery, Inselspital, Bern University Hospital and University of Bern, Bern, Switzerland; ^2^ Department of Radiation-Oncology, Inselspital, Bern University Hospital and University of Bern, Bern, Switzerland; ^3^ Department of Otorhinolaryngology, Head and Neck Surgery, University Hospital Heidelberg, Heidelberg, Germany; ^4^ Department of Otorhinolaryngology, Head and Neck Surgery, CHUV, University of Lausanne, Lausanne, Switzerland

**Keywords:** morbidity, paranasal sinus cancer, endoscopic surgery, mortality, nasal cavity cancer

## Abstract

**Introduction:**

Sinonasal malignancies are rare and histologically heterogeneous cancers of the nasal cavity and sinuses. The treatment of choice is usually surgery and, if necessary, adjuvant radiotherapy. In this study, we aimed to investigate treatment modalities and associated morbidity.

**Methods:**

A consecutive case series of solid sinonasal cancer treated at our tertiary referral center was analyzed. We performed a retrospective chart review and statistical analysis.

**Results:**

A total of 156 patients with sinonasal cancer were enrolled in the present study. Male patients were more frequently affected (62%) and the median age was 64 years. Squamous cell carcinoma, adenocarcinoma and malignant melanoma (MM) were the most common histopathological entities. Surgery was the primary treatment modality for 73% of curatively treated patients. Primary radiotherapy alone or in combination with systemic treatment was less frequent. Median overall (OS) and recurrence-free survival (RFS) was 164 months and 71.3 months, respectively. Multivariate analysis revealed negative associations of histology (MM) and skull base involvement on RFS and age, skull base involvement and the type of primary therapy (radiochemotherapy) on OS. Postoperative 30-day morbidity was low, with most patients (84%) experiencing no reported events. Radiotherapy was generally well-tolerated, despite most of patients experienced acute toxicity such as dermatitis (80.6%) or mucositis (72.1%). However, only one event of acute toxicity > grade 3 was reported. Long term morbidity was most frequently reported as pain (23%), dry mucosa (19%) and anosmia (14%).

**Conclusion:**

We observed negative associations of histology (MM) and skull base involvement on RFS and age, skull base involvement and the type of primary therapy (radiochemotherapy) on OS. Acute treatment-related morbidity was generally low for surgical patients and considerable for irradiated patients. Moreover, a consistent part of the cohort displayed long term morbidity.

## 1 Introduction

Sinonasal malignancies are a rare group of heterogeneous histopathological cancers located in the nasal cavity and paranasal sinuses. They are estimated with a low incidence of 0.5-1 per 100’000 in the general population (1, 2) and vary greatly in etiology and prognosis (3). Among those entities, the most common histopathological findings are squamous cell carcinoma (SCC), adenocarcinoma (AC) and malignant melanoma (MM) (4, 5).

In contrast to the heterogeneity of entities, treatment for sinonasal cancer is rather uniform: Surgery is considered the modality of choice in non-metastatic disease (6), with endoscopic surgery having been demonstrated to be non-inferior to open surgery while reducing complications (4, 7). Furthermore, (chemo) radiotherapy can be applied in the adjuvant or definitive setting, with promising results in terms of survival (8). However, sinonasal tumors initially often present with non-specific symptoms or may even be asymptomatic. Consequently, tumors are regularly diagnosed with an advanced stage at first presentation (9–11). Therefore, a combination of different treatment modalities may be warranted by the interdisciplinary tumor board, especially in advanced stages or situations with a high risk for R1- or R2-resection (12). The proximity to vital structures such as the skull base, orbit and airway is poses additional challenges in the management of sinonasal malignancies (13).

However, there is a lack of evidence on the treatment-related morbidity to guide decision-making (14). Morbidity after treatment of sinonasal tumors typically includes general rhinological symptoms such as epistaxis, crusting or impaired nasal breathing, but also more treatment-related complications such as flap necrosis or radiation toxicity. The aim of this study was to further investigate the treatment-related morbidity of curatively treated patients with sinonasal malignancies. Together with treatment- and survival-related data, these findings will be important for counselling and determining individualized treatment strategies.

## 2 Materials and methods

### 2.1 Ethical considerations

All procedures were in accordance with the ethical standards of the national research committee and with the 1964 and 2002 Helsinki declaration. The institutional and regional review board (Inselspital, Bern University Hospital, Switzerland, reference number KEK-BE 002/2015) granted approval to conduct the study.

### 2.2 Patients and data acquisition

A retrospective chart review was conducted. Patients with histologically confirmed solid cancer of the nose and/or paranasal sinuses discussed at the multidisciplinary tumor board of the Head and Neck Cancer Center, Inselspital, Bern University Hospital, were included. Exclusion criteria were non-solid tumors such as lymphoma or nasal vestibule location, and loss to follow-up after treatment. Data were extracted from reports of the Head and Neck Cancer Center. Collected information included patient characteristics, TNM classification (according to the Union for International Cancer Control, TNM Classification 7th edition, 2010), treatment modalities and reported findings of the routine follow-up consultations concerning morbidity.

### 2.3 Statistical analysis

Statistical Analysis was performed using R (v 4.1.2). Except where explicitly stated, patients treated with a palliative concept were excluded from inferential statistics. For multivariable models, we pre-selected variables based on likely clinical significance: tumor location, UICC/TNM stage, resection margin (R), age, sex, histology, involvement of the skull base, smoking, primary therapy modality, alcohol abuse, smoking and tumor grading (G). Variables with >50% missing values were excluded from imputation and analysis. Missing values were imputed using the MICE (v 3.16.0) package using standard options. Next, we performed multivariable regression with two separate feature selection techniques to increase confidence for selected variables. For stepwise variable elimination, the MASS (v7.3-54) package was used with both in- and exclusion, based on the Akaike information criterion (AIC). The second procedure was LASSO via package glmnet (v 4.1-8), using best lambda (smallest cross-validated error). Variables identified by stepwise selection or LASSO then entered a multivariable Cox regression model with the chosen endpoint. Concerning toxicity, out of all recorded symptoms, only the ones with at least one event > grade 1 in the cohort are reported. For radiotherapy, late toxicities are defined as occurring >90 days after the last applied fraction. Regarding endpoints, we defined recurrence-free survival (RFS) events as having any tumor recurrence (local, regional, distant) or death. Patients without event were censored at the time of last follow-up.

## 3 Results

### 3.1 Patient and tumor characteristics

We identified 156 eligible patients, treated at our institution, between 2008 and 2023 as summarized in **Table 1**. The median follow-up was of 41.5 months. The median age at diagnosis was 64 (range 9 – 92) years with 62% male and 38% female patients. Profession was unknown in 94 cases, but among those with available data, we identified 19% having worked as carpenters. Concerning oncological disease, 51% were seated in the nasal cavity and 34% in the paranasal sinuses. The remaining 15% of cases had tumors that overlapped the boundaries between two anatomical regions. Invasion of the base of skull was present in 28% of patients. Tumor size at diagnosis trended towards higher T stages with 15% T3 and 49% T4 lesions. Clinical nodal stage (cN) was zero in the majority of patients (89%) and there were few N1-2, and no N3 cases in our cohort. Distant metastases were present only in 4% of patients at the time of diagnosis. Histopathologically, eight different entities were identified, most commonly squamous cell carcinoma (40%), adenocarcinoma (17%) and melanoma (15%). Regarding adenocarcinoma histology, we observed intestinal (58%) and non-intestinal (27%) subtypes respectively. In 4 patients, the histology was not further specified (15%). Histologically, few tumors were grade 1 (7%), compared to 31% grade 2 and 26% grade 3 lesions. However, almost a third of patients had inconclusive grading and were classified as Gx.

**Table 1:** Patient and tumor characteristics.

Characteristics	N = 156*^1^*
Median age	64 (53, 75)
Male sex	97 (62%)
Primary tumor location	
* nasal cavity*	80 (51%)
* paranasal sinuses*	53 (34%)
* overlapping*	23 (15%)
T Stage	
* 1*	35 (22%)
* 2*	20 (12%)
* 3*	23 (15%)
* 4a*	46 (30%)
* 4b*	30 (19%)
* x*	2 (1.3%)
cN Stage	
* 0*	139 (89%)
* 1*	9 (5.9%)
* 2a*	2 (1.3%)
* 2b*	3 (2.0%)
* 2c*	3 (2.0%)
pN Stage	
* 0*	13 (76%)
* 1*	3 (18%)
* 2b*	1 (5.9%)
M Stage	
* 0*	147 (94%)
* 1*	6 (3.9%)
* x*	3 (1.9%)
Histology	
* squamous cell carcinoma*	62 (40%)
* adenocarcinoma*	26 (17%)
* mucosal melanoma*	24 (15%)
* adenoid cystic carcinoma*	10 (6.4%)
* esthesioneuroblastoma*	10 (6.4%)
* sinonasal undifferentiated carcinoma*	6 (3.8%)
* sarcoma*	5 (3.2%)
* other*	13 (8.3%)

^1^Median (IQR); n (%).

### 3.2 Therapy

A total of 141 patients (90%) were treated with curative intent while the remaining 15 individuals were referred for a palliative approach (e.g., debulking surgery, palliative radiotherapy or systemic therapy). In curatively treated patients, surgery was chosen as the primary modality in 73% of cases, while radiotherapy alone (10%) or in combination with a systemic agent (17%) was less common as summarized in **Table 2**. Extensive local surgery (open or open and endoscopic combined) was performed in 30% of cases, including orbital exenteration, nasal ablation and skull base resection as specified in **Table 2**. Neck dissection was performed in 12% of cases and reconstructive procedures such as tissue flaps and/or epithetic protheses were used in 12% of surgical patients. A histopathologically equivocal resection margin (Rx) was seen in 49% of patients after surgery. In the remaining 51% of patients R0, R1 and R2 resection was performed in 22%, 24% and 5% respectively. As a result, primary surgery was frequently followed by adjuvant radiotherapy (68 cases) to treat high-risk areas, either alone or along with systemic therapy. Photon beam radiotherapy was the most common modality (80%) among irradiated patients. In the remaining patients, 19% were treated with proton beam radiotherapy and one patient was treated with carbon ion radiotherapy. The vast majority of patients (97%) were treated with intensity-modulated radiotherapy (IMRT). In 75% of patients, a local radiotherapy including the primary tumor bed was performed. Twenty-four percent of the patients received a locoregional radiotherapy, including the primary tumor and the regional lymphatic drainage pathways with or without lymph node metastases. Only one patient was treated with regional radiotherapy only.

**Table 2:** Characteristics of primary curatively intended treatment (N=141).

Characteristics	SCC N = 56*^1^*	AC N = 25*^1^*	MM N = 21*^1^*	Other N = 39*^1^*
Primary therapy				
* surgery*	30 (54%)	23 (92%)	19 (90%)	31 (79%)
* radiochemotherapy*	17 (30%)	1 (4.0%)	1 (4.8%)	5 (13%)
* radiotherapy*	9 (16%)	1 (4.0%)	1 (4.8%)	3 (7.7%)
Resection margins				
* R0*	9 (16%)	9 (36%)	6 (29%)	7 (18%)
* R1*	9 (16%)	4 (16%)	6 (29%)	15 (38%)
* R2*	2 (3.6%)	2 (8.0%)	2 (9.5%)	1 (2.6%)
* Rx*	36 (64%)	10 (40%)	7 (33%)	16 (41%)
Nose ablation	7 (13%)	1 (4.0%)	3 (14%)	0 (0%)
Orbita exenteration	8 (14%)	1 (4.0%)	4 (19%)	3 (7.7%)
Skullbase resection	1 (1.8%)	6 (24%)	1 (4.8%)	8 (21%)
Neck dissection	6 (11%)	3 (12%)	4 (19%)	4 (10%)
Reconstruction				
* epithesis*	1 (1.8%)	0 (0%)	1 (4.8%)	0 (0%)
* epithesis and free flap*	1 (1.8%)	0 (0%)	0 (0%)	0 (0%)
* free flap*	4 (7.1%)	1 (4.0%)	3 (14%)	5 (13%)
* local flap*	0 (0%)	0 (0%)	0 (0%)	1 (2.6%)
* none*	50 (89%)	24 (96%)	17 (81%)	33 (85%)
RT Delivered Dose	70 (47, 74)	66 (54, 72)	66 (18, 70)	66 (39, 74)
RT Fraction Dose	2.00 (1.80, 4.00)	2.00 (1.80, 2.00)	2.00 (2.00, 3.00)	2.00 (1.80, 3.00)
RT Target Region				
* primary*	27 (61%)	16 (94%)	16 (89%)	22 (76%)
* both*	17 (39%)	1 (5.9%)	1 (5.6%)	7 (24%)
* neck*	0 (0%)	0 (0%)	1 (5.6%)	0 (0%)
Chemotherapy				
* adjuvant*	9 (16%)	2 (8.0%)	6 (29%)	2 (5.1%)
* concomitant*	9 (16%)	0 (0%)	1 (4.8%)	4 (10%)
* multiple*	5 (8.9%)	1 (4.0%)	0 (0%)	0 (0%)
* neoadjuvant*	5 (8.9%)	1 (4.0%)	0 (0%)	2 (5.1%)
* none*	28 (50%)	21 (84%)	14 (67%)	31 (79%)

^1^n (%); Median (Range), SCC, squamous cell carcinoma; AC, adenocarcinoma; MM, mucosal melanoma.

### 3.3 Complications and toxicity

Information about postoperative 30-day morbidity was available in 70 cases, 84% of which had no reported events. Out of the remaining 11 cases with complications (**Table 3**), infection was the most frequent postoperative morbidity (6 cases) followed by nose bleeding in 4 cases. Six patients had to be hospitalized with the most common treatments being a surgical intervention and antibiotic treatment (**Table 3A**). Clavien-Dindo score was not reported for most patients, but was distributed between 1 – 4a without any grade 5 (death) events. Radiotherapy was generally well-tolerated by the 119 patients with available data, demonstrating no > grade 3 event except one acute grade 5 pulmonary aspiration potentially related to treatment (**Table 3B**). Acute dermatitis (80.6%), acute mucositis (72.1%) followed by acute dysgeusia (36%) and acute fatigue (33.9%) were the most common radiotherapy-related morbidities. The morbidity by last follow-up for all patient groups is presented in **Table 4**. Pain (23%), dry nasal mucosa (19%), recurrent epistaxis (14%), nasal obstruction (14%), nasal crusting (14%), anosmia (14%) and orbital exenteration (14%) were the most frequent long-term morbidities at the last follow-up.

**Table 3:** Acute treatment-related morbidity.

3A Surgical morbidity including revision surgery		
	Endoscopic exclusive N = 61*^1^*	Combined and open N = 49*^1^*
Acute morbidity (30 days after surgery)		
* bleeding*	2 (4.3%)	2 (8.3%)
* flap insufficiency*	0 (0%)	1 (4.2%)
* infection*	4 (8.7%)	1 (4.2%)
* none*	39 (85%)	20 (83%)
* skin erythema*	1 (2.2%)	0 (0%)
* unknown*	15	25
Treatment of 30-day morbidity		
* surgery*	3 (60%)	1 (33%)
* antibiotic*	2 (40%)	1 (33%)
* hospitalization*	4 (80%)	2 (67%)

3B Radiotherapy-related morbidity		N=106
Acute dysphagia		
* 1*		9
* 2*		9
* 3*		4
Acute mucositis		
* 1*		29
* 2*		29
* 3*		10
Acute fatigue		
* 1*		30
* 2*		1
* 3*		1
Acute pain		
* 1*		9
* 2*		17
* 3*		2
Acute dermatitis		
* 1*		52
* 2*		18
* 3*		6
Acute xerostomia		
* 1*		19
* 2*		8
* 3*		1
Acute conjunctivitis		
* 1*		12
* 2*		3
* 3*		2
Acute dysgeusia		
* 1*		20
* 2*		12
* 3*		2

Number 1-3 relate to grade of toxicity.

**Table 4:** Morbidity per last follow-up, sorted by frequency (all treatment modalities, data available for n=118 patients).

Characteristic	Whole cohort N= 118	Endoscopic surgery N = 61*^1^*	Combined or open N = 49*^1^*
Pain	27 (23%)	13 (21%)	10 (20%)
Dry endonasal mucosa	22 (19%)	10 (16%)	10 (20%)
Anosmia	17 (14%)	8 (13%)	7 (14%)
Recurrent epistaxis	16 (14%)	7 (11%)	7 (14%)
Orbita exenteration	16 (14%)	2 (3.3%)	14 (29%)
Impaired nasal breathing	16 (14%)	9 (15%)	6 (12%)
Nasal crusting	16 (14%)	11 (18%)	5 (10%)
Dacryo-cysto-stenosis	13 (11%)	6 (9.8%)	4 (8.2%)
Nose ablation	11 (9.3%)	2 (3.3%)	9 (18%)
Blindness	11 (9.3%)	4 (6.6%)	6 (12%)
Chronic rhinorrhea	8 (6.8%)	6 (9.8%)	1 (2.0%)
Middle ear problem	8 (6.8%)	3 (4.9%)	5 (10%)
Xerostomia	5 (4.2%)	2 (3.3%)	3 (6.1%)
Feeding tube	4 (3.4%)	0 (0%)	4 (8.2%)
Plegia or paresis	4 (3.4%)	3 (4.9%)	1 (2.0%)
Fatigue	3 (2.5%)	0 (0%)	3 (6.1%)
Recurrent sinusitis	3 (2.5%)	1 (1.6%)	2 (4.1%)
Chronic nausea	3 (2.5%)	2 (3.3%)	1 (2.0%)
Paraesthesia	3 (2.5%)	1 (1.6%)	2 (4.1%)
Tracheotomy	3 (2.5%)	1 (1.6%)	2 (4.1%)
Glossitis	1 (0.8%)	1 (1.6%)	0 (0%)

^1^n (%). In the endoscopic or combined/open surgery groups adjuvant radiotherapy is included.

### 3.4 Oncological outcome

**Figure 1:**
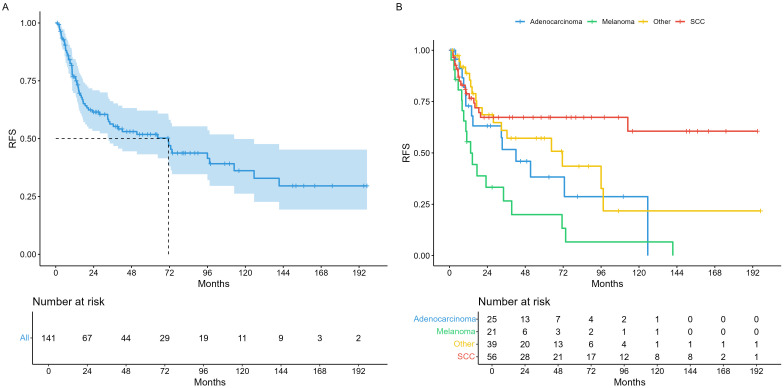
Recurrence-free survival. **(A)** Whole cohort, **(B)** per histological entities.

**Figure 2:**
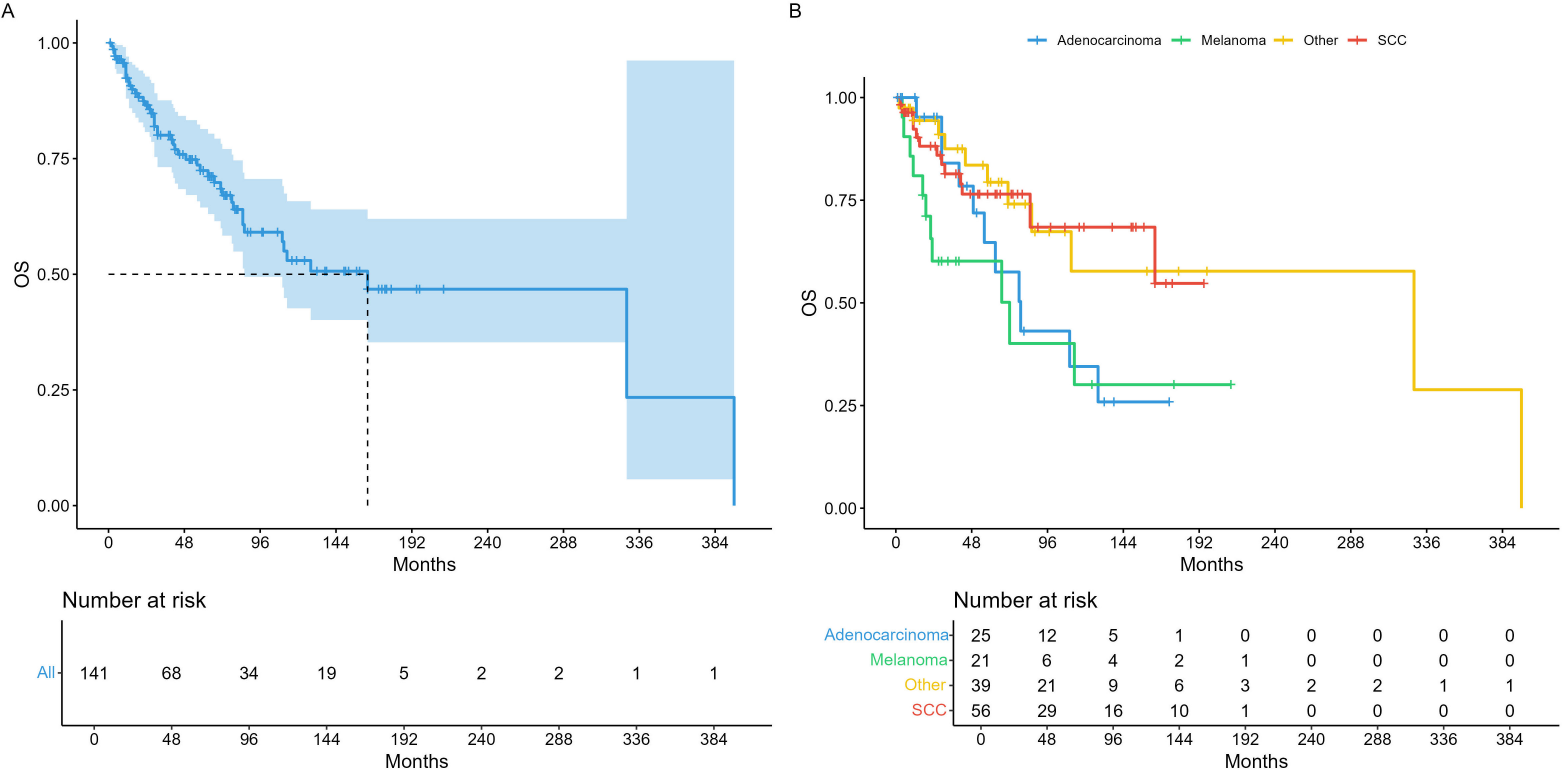
Overall survival. **(A)** Whole cohort, **(B)** per histological entities.

Median survival times in the entire cohort were 71.3 months recurrence-free survival (RFS) as illustrated in **Figure 1A**. For analysis of RFS in the histopathological subgroups, we divided tumors in the three most prevalent groups (SCC, AC, MM), and a fourth group comprising all other histologies (OTH) as shown in **Figure 1B**. Median overall survival (OS) was 164 months for the whole cohort (**Figure 2A**) and illustrated in the same histopathological subgroups in **Figure 2B**. In the 66 patients with any recurrence or persistence of disease, 47% experienced only local failure, followed by distant (17%) and regional (11%) recurrence. The remaining 25% of patients had multiple sites of recurrence (e.g., simultaneously local and distant, loco-regional etc.). Univariable Cox regression of RFS with AC as baseline (HR = 1) showed a statistically significant difference for SCC (HR = 0.44, 95% CI 0.21 – 0.90), but not for MM (HR = 1.87, 95% CI 0.93 – 3.78) or OTH (HR = 0.72, 95% CI 0.36 – 1.47). For OS, no individual histology was shown to be statistically significant, but the global model p-value reached p = 0.038.

In multivariable regression with endpoint RFS, the model without selection identified histology, smoking and alcohol abuse as statistically significant covariates (**Table 5**). Out of these variables, LASSO and stepwise selection both confirmed histology, but additionally revealed an association with skull base involvement. (**Supplementary Table S1**). With endpoint OS, age, skull base involvement and the type of primary therapy were shown to be statistically significant covariates (**Table 5**). In summary, we observed negative associations of MM histology and skull base involvement on RFS and age, skull base involvement and the type of primary therapy (radiochemotherapy) on OS. When employing LASSO or stepwise selection, the two resulting models differed somewhat in the number of included variables, but agreed with the unselected model in terms of statistically significantly associated parameters (**Supplementary Table S2**). The chemotherapy regimens showed a high variability and are summarized in **Supplementary Table S3**.

**Table 5:** Multivariable Cox regression, endpoints RFS and OS.

	RFS			OS		
Characteristic	HR*^1^*	95% CI*^1^*	p-value	HR*^1^*	95% CI*^1^*	p-value
Alcohol abuse	0.77	0.62, 0.95	0.013	1.18	0.89, 1.55	0.2
Primary tumor location			0.2			0.069
* nasal cavity*	—	—		—	—	
* overlapping*	0.47	0.19, 1.15		0.76	0.27, 2.12	
* paranasal sinuses*	1.01	0.51, 2.02		2.52	1.05, 6.00	
Stage			0.2			0.2
* I*	—	—		—	—	
* II*	0.41	0.11, 1.49		1.36	0.15, 12.3	
* III*	0.45	0.15, 1.32		4.49	0.70, 28.9	
* IV*	0.96	0.35, 2.62		1.83	0.30, 11.3	
Resection margins			0.062			0.081
* R0*	—	—		—	—	
* R1*	1.31	0.51, 3.40		1.16	0.29, 4.66	
* R2*	1.42	0.32, 6.35		4.40	0.91, 21.3	
* Rx*	3.02	1.22, 7.51		0.72	0.20, 2.61	
Age	0.99	0.97, 1.01	0.3	1.05	1.01, 1.08	0.006
Sex			0.2			>0.9
* female*	—	—		—	—	
* male*	0.63	0.32, 1.23		1.04	0.39, 2.77	
Histology			<0.001			0.087
* adenocarcinoma*	—	—		—	—	
* SCC*	0.33	0.13, 0.83		0.63	0.17, 2.31	
* melanoma*	6.58	1.78, 24.4		3.33	0.58, 19.0	
* other*	0.96	0.38, 2.40		0.66	0.17, 2.47	
Skull base involvement			0.2			0.014
* no*	—	—		—	—	
* yes*	1.77	0.80, 3.95		3.56	1.27, 9.92	
Grade			0.3			0.3
* 1*	—	—		—	—	
* 2*	1.40	0.42, 4.61		1.27	0.20, 7.92	
* 3*	3.26	0.97, 10.9		3.26	0.47, 22.8	
* 4*	1.76	0.39, 7.88		3.69	0.27, 51.2	
*x*	1.39	0.46, 4.24		1.31	0.16, 10.5	
Smoking			0.008			0.10
* never*	—	—		—	—	
* ongoing*	3.63	1.35, 9.74		0.62	0.18, 2.09	
* past*	4.69	1.65, 13.3		2.02	0.59, 7.01	
Primary therapy			>0.9			0.006
* radiochemotherapy*	—	—		—	—	
* radiotherapy*	1.00	0.29, 3.48		1.29	0.28, 5.84	
* surgery*	1.10	0.39, 3.10		0.18	0.05, 0.61	

^1^HR, Hazard Ratio; CI, Confidence Interval.

## 4 Discussion

In this study, 156 patients with sinonasal cancer were included to investigate the different treatment modalities and their morbidity. The majority of patients were diagnosed with SCC and advanced local tumor stage (T3-T4 64%), whereas regional lymph node metastases were rare (cN+ 11%). Curative treatment was possible for 141 patients. Similarly, Bracigliano et al. (2021) have recently described a large number of histological subtypes, with SCC being the most common entity (15). The increased use of genetic analysis has led to the identification of new subtypes of histopathological entities, which have not influenced treatment strategies so far. According to the current literature, treatment modalities include surgery, radiotherapy, chemotherapy, or a combination of these (5, 12). This treatment strategy is recommended despite the wide variety of histological entities (15). An exception is MM where protocols using targeted or immunotherapy have been described in the therapy of mucosal melanomas with promising results (16, 17).

The overall 5-year survival vary markedly depending on the histological subtype between 20-70% (18). It has been observed that patients diagnosed with esthesioneuroblastoma or chondrosarcoma tend to have a more favorable outcome, while those with undifferentiated sinonasal or NUT carcinoma often face more challenging circumstances (19). Consequently, some authors propose categorization into different risk groups, particularly in order to adapt follow-up care to the corresponding biological characteristics (11). Carey et al. (2017) analyzed a large cohort of patients with sinonasal squamous cell carcinomas between 2004-2012 with a median survival of 53.4 months (20). Recently, Schur et al. (2024) describes in his cohort a median survival of 76 months with curative therapy and a 5-year OS of 57% in locally advanced sinonasal cancer (21). The median overall survival reported in our cohort is considerably longer with 164 months; however, all tumor types and stages were included in our study. Moreover, the ethnic background of the patient appears to be a relevant factor with regard to the prognosis. Several studies indicating that black ethnicity may experience a less favorable prognosis compared to their caucasian counterparts (22).

In recent years, an increasing number of studies have been published that favor endoscopic tumor resection over open resection (23, 24). It has been demonstrated that endoscopic surgery results in less morbidity with similar oncological outcome (21, 25). However, depending on the extent of the tumor, open surgical approaches are sometimes unavoidable. Of the surgically treated patients in this cohort, in 30 cases (29%), extended surgery was required with nasal amputation, orbital exenteration or skull base resection. Since exenteration of the orbit and amputation of the nose lead to severe aesthetic and functional deterioration in the quality of life and psychological consequences, this surgery should be reserved for extensive tumors with infiltration of these critical structures (26). To achieve optimal patient selection and avoid unnecessarily radical surgery, Ferrari et al. (2021) have developed an overview with the most relevant anatomical structures that should be considered when selecting the appropriate surgical procedure (27). Some authors recommend that a systematic intraoperative assessment should be carried out prior to definitive treatment to evaluate the extent of infiltration of these structures (28).

Regional metastases are uncommon in sinonasal cancer (29). Therefore, treatment of the neck lymph nodes is typically performed in the clinical N+ stage or in selected situations, although there is no clear consensus on this question (23, 27). Of the surgically treated patients in this cohort, only 17 (16.5% of all surgeries) underwent neck dissection.

Concerning preoperative therapy, several clinical studies have been initiated, testing the benefit of induction chemotherapy in sinonasal malignancies. One example is the SINTART 1 study that investigated whether preoperative treatment with up to five cycles of chemotherapy, followed by surgery or (chemo-)radiotherapy, was feasible and safe (30). In the resulting publication, the authors describe a median progression-free survival (PFS) of 26 months and a five-year overall survival of 46%. The objective response rate across all included histologies was 54% with 9% complete responses, overall. This work was followed by the SINTART 2 trial, testing histology-adapted induction chemotherapy with MRI-based response assessment and radiotherapy (photons, protons or carbon ions) with or without concurrent chemotherapy (31). Compared to its predecessor, median PFS was lower at 18 months, as was five-year OS at 23.8%. There was a numerical but no statistically significant PFS- and OS-benefit for patients whose tumors showed a major volumetric partial response, therefore conclude that their approach was not successful. A newer study of induction chemotherapy in advanced and poorly differentiated disease reported encouraging results with a median disease-free survival and OS of 19.2 and 47.4 months, respectively, but final results are pending (32).

Adjuvant radiotherapy was administered in the 62% of cases where clear margins could not be achieved, patho-histological risk factors were present, or the tumor was at an advanced stage. The preferred technique was IMRT in 92% of cases with a median dose of 66 Gy. This highly conformal technique has been the gold standard for a number of years now, as it causes fewer side effects in critical organs at risk (e.g., orbit, cochlea, brain) (33, 34). Primary radiotherapy or combined chemoradiotherapy was performed in the 25.6% of cases if surgery was not feasible or refused by the patient. Acute side effects of radiotherapy included mucositis, dermatitis, conjunctivitis, dysphagia and pain. Severe side effects were rarely observed, the most common being mucositis and dermatitis. Compared with Askoxylakis et al. (2016), the number of severe acute side effects was slightly higher (35). This could be due to the higher total dose used in our setting (66 versus 64 Gy). Additionally, they described five cases of acute and eight cases of late visual impairment (32), which is similar to our data. They suggested an association with the maximum total dose applied to the eye, but not to the optic nerve nor to the optic chiasm. Similarly, observed morbidity in SCC patients correlates with disease extension, as a more aggressive or multimodal treatment regime can be avoided in less advanced stages (27). A recently published study by Levin et al. (2023) describes other ocular problems besides visual impairment following radiotherapy, such as keratoconjunctivitis sicca, retinal detachment, lacrimal duct pathologies, cataract and pain (13). In addition to ocular side effects, sinusitis and endonasal synechiae were frequently observed and even may have required surgical intervention. A variety of other radiotherapy-related side effects such as osteonecrosis, neuropathy and dysphagia have been reported.

Multivariable Cox models revealed several relevant factors that were associated with time-to-event outcomes. Concerning histology, melanoma demonstrated a markedly higher risk for RFS (HR 6.58) than the baseline, AC. In contrast, the hazard ratio for SCC was much lower (HR 0.3), but this did not translate to a statistically significant improved OS. Another relevant parameter was skull base involvement, which influenced OS but not RFS, most likely reflecting the generally worse prognosis of more advanced tumors. Similarly, the use of (chemo)radiotherapy as primary treatment was associated with a decreased OS, but the absence of this difference in RFS suggests that it was likely caused by its predominant use in inoperable patients with a worse overall prognosis. Interestingly, resection margins did not have a statistically significant effect on RFS with the exception of Rx. One reason could be that R1 and R2 resections automatically trigger adjuvant treatment, which might have mitigated this disadvantage.

This study has limitations by its retrospective nature and the lack of the evaluating the morbidity with a validated questionnaire such as the Anterior Skull Base Surgery Questionnaire (ASBS-Q). In this study, we used the morbidity as reported by the patient during clinical follow-ups. However, some morbidities may have been missed. Moreover, we were unable to differentiate the morbidity caused by the tumor versus treatment-related morbidities. Finally, we acknowledge a relatively small cohort size, especially concerning the different histological entities and treatment modalities.

In conclusion, we report disease control and overall survival in a diverse variety of sinonasal malignancies. Despite intensive, often multimodal treatment, side effects appeared tolerable in the short and long term, and severe post-therapeutic complications are rare. There was a discernable effect of histology on the risk of disease recurrence, with melanoma conferring the worst, and SCC the best prognosis, respectively.

## Data Availability

The raw data supporting the conclusions of this article will be made available by the authors, without undue reservation.
