# Supplementary material for: Treatment and related morbidity of nasal cavity and paranasal sinus cancers
Source: Front Oncol. 2024 Sep 26;14:1422892. doi: 10.3389/fonc.2024.1422892 (PMC11464990; doi:10.3389/fonc.2024.1422892)
Supplement: Supplementary file 1 [file Table1.docx]

Supplementary Material

Treatment and Related Morbidity of Nasal Cavity and Paranasal Sinuses Cancers

**Nils Smaadahl, MD^1*^; Sara-Lynn Hool, MD^1*^; Philipp Reinhardt, MD^2^; Lucas Mose, MD^2^; Ralph Hohenberger, MD^3^; Roland Giger, MD^1^; Daniel Henrik Schanne, MD^2°^; Lukas Anschuetz, MD^1°^**

*** Correspondence:** Roland Giger [roland.giger@insel.ch](mailto:roland.giger@insel.ch)

# Supplementary Figures and Tables

**Table S1:** Multivariable Cox regression, endpoint RFS, LASSO and stepwise variable selection

|  | **Stepwise Selection** | | | **LASSO selection** | | |
| --- | --- | --- | --- | --- | --- | --- |
| **Characteristic** | **HR***^1^* | **95% CI***^1^* | **p-value** | **HR***^1^* | **95% CI***^1^* | **p-value** |
| Alcohol abuse | 0.86 | 0.72, 1.03 | 0.10 |  |  |  |
| Histology |  |  | <0.001 |  |  | <0.001 |
| *adenocarcinoma* | — | — |  | — | — |  |
| *melanoma* | 3.52 | 1.49, 8.34 |  | 2.27 | 1.10, 4.70 |  |
| *other* | 0.94 | 0.44, 2.01 |  | 0.73 | 0.36, 1.48 |  |
| *SCC* | 0.55 | 0.25, 1.17 |  | 0.52 | 0.25, 1.08 |  |
| Skull base involvement |  |  | 0.007 |  |  | 0.023 |
| *no* | — | — |  | — | — |  |
| *yes* | 2.35 | 1.30, 4.26 |  | 2.00 | 1.13, 3.57 |  |
| Smoking |  |  | 0.11 |  |  |  |
| *never* | — | — |  |  |  |  |
| *ongoing* | 2.25 | 0.96, 5.26 |  |  |  |  |
| *past* | 2.29 | 1.0, 5.29 |  |  |  |  |
| *^1^*HR = Hazard Ratio, CI = Confidence Interval | | | | | | |

**Table S2:** Multivariable Cox regression, endpoint OS, LASSO and stepwise variable selection

|  | **Stepwise Selection** | | | **LASSO selection** | | |
| --- | --- | --- | --- | --- | --- | --- |
| **Characteristic** | **HR***^1^* | **95% CI***^1^* | **p-value** | **HR***^1^* | **95% CI***^1^* | **p-value** |
| Primary tumor location |  |  | 0.068 |  |  | 0.064 |
| *nasal cavity* | — | — |  | — | — |  |
| *overlapping* | 0.61 | 0.23, 1.65 |  | 0.76 | 0.27, 2.12 |  |
| *paranasal sinuses* | 2.21 | 0.97, 5.03 |  | 2.53 | 1.08, 5.96 |  |
| Stage |  |  | 0.055 |  |  | 0.2 |
| *I* | — | — |  | — | — |  |
| *II* | 1.84 | 0.22, 15.5 |  | 1.36 | 0.15, 12.1 |  |
| *III* | 7.31 | 1.24, 43.1 |  | 4.47 | 0.70, 28.5 |  |
| *IV* | 2.81 | 0.53, 15.0 |  | 1.81 | 0.30, 11.1 |  |
| Resection margins |  |  | 0.054 |  |  | 0.057 |
| *R0* | — | — |  | — | — |  |
| *R1* | 0.91 | 0.26, 3.16 |  | 1.16 | 0.29, 4.62 |  |
| *R2* | 4.00 | 0.97, 16.4 |  | 4.42 | 0.92, 21.3 |  |
| *Rx* | 0.66 | 0.21, 2.11 |  | 0.71 | 0.20, 2.47 |  |
| Age | 1.04 | 1.01, 1.07 | 0.007 | 1.05 | 1.01, 1.08 | 0.006 |
| Histology |  |  | 0.074 |  |  | 0.085 |
| *adenocarcinoma* | — | — |  | — | — |  |
| *melanoma* | 3.86 | 1.05, 14.2 |  | 3.36 | 0.60, 19.0 |  |
| *other* | 0.89 | 0.31, 2.59 |  | 0.66 | 0.18, 2.44 |  |
| *SCC* | 0.80 | 0.24, 2.65 |  | 0.64 | 0.18, 2.27 |  |
| Skull base involvement |  |  | <0.001 |  |  | 0.012 |
| *no* | — | — |  | — | — |  |
| *yes* | 4.62 | 1.85, 11.6 |  | 3.58 | 1.31, 9.82 |  |
| Smoking |  |  | 0.061 |  |  | 0.065 |
| *never* | — | — |  | — | — |  |
| *ongoing* | 1.13 | 0.44, 2.92 |  | 0.62 | 0.18, 2.09 |  |
| *past* | 3.02 | 1.14, 8.04 |  | 2.06 | 0.66, 6.44 |  |
| Primary therapy |  |  | 0.005 |  |  | 0.005 |
| *radiochemotherapy* | — | — |  | — | — |  |
| *radiotherapy* | 1.36 | 0.35, 5.24 |  | 1.28 | 0.29, 5.71 |  |
| *surgery* | 0.19 | 0.06, 0.57 |  | 0.17 | 0.05, 0.57 |  |
| Alcohol abuse |  |  |  | 1.18 | 0.89, 1.55 | 0.2 |
| Grade |  |  |  |  |  | 0.3 |
| *1* |  |  |  | — | — |  |
| *2* |  |  |  | 1.27 | 0.20, 7.93 |  |
| *3* |  |  |  | 3.26 | 0.47, 22.8 |  |
| *4* |  |  |  | 3.64 | 0.27, 49.5 |  |
| *x* |  |  |  | 1.29 | 0.17, 9.97 |  |
| *^1^*HR = Hazard Ratio, CI = Confidence Interval | | | | | | |

**Table S3:** Substances for systemic therapies

| Neoadjuvant |
| --- |
| *carboplatin/docetaxel* |
| *carboplatin/docetaxel/5-FU* |
| *carboplatin/paclitaxel* |
| *cisplatin/docetaxel* |
| *cisplatin/docetaxel/5-FU* |
| *cisplatin/docetaxel/capecitabine* |
| *doxorubicin* |
| Adjuvant |
| *ipilimumab/nivolumab* |
| *nivolumab* |
| *pembrolizumab* |
| Concomitant |
| *carboplatin/5-FU* |
| *carboplatin/paclitaxel* |
| *cetuximab* |
| *cisplatin* |
| *cisplatin/docetaxel* |
| *cisplatin/etoposide* |
| *nivolumab* |
